# We12BFit!—Improving Physical Fitness in 7–12-Year-Old Children With Developmental Coordination Disorder: Protocol of a Multicenter Single-Arm Mixed-Method Study

**DOI:** 10.3389/fped.2018.00396

**Published:** 2018-12-18

**Authors:** Petra Braaksma, Ilse Stuive, Frouwien D. van der Hoek, Corry K. van der Sluis, Marina M. Schoemaker, Rienk Dekker

**Affiliations:** ^1^Department of Rehabilitation Medicine, University Medical Center Groningen, University of Groningen, Groningen, Netherlands; ^2^Center for Rehabilitation, University Medical Center Groningen, University of Groningen, Groningen, Netherlands; ^3^Center for Human Movement Sciences, University Medical Center Groningen, University of Groningen, Groningen, Netherlands

**Keywords:** child, motor skills disorders, developmental coordination disorder (DCD), physical fitness, intervention studies, treatment theory

## Abstract

**Background:** Children with developmental coordination disorder (DCD) are less physically fit than their typically developing peers. No substantiated treatments are available for children with DCD to address this issue.

**Aims:** This study aims to describe 1. the design and rationale of We12BFit!-PF, a training to increase cardiorespiratory fitness, muscle strength and anaerobic power in 7-12-year-old children with DCD and 2. the methods to examine its preliminary effectiveness and feasibility.

**Methods:** We12BFit!-PF was developed using the steps of defining a treatment theory as proposed by Whyte et al. This includes the definition of targets, mechanisms of action, and essential ingredients. We12BFit!-PF will be evaluated in children diagnosed with DCD according to the criteria of the Diagnostic and Statistical Manual of mental disorders (DSM-V) aged 7–12, recruited from rehabilitation centers and physical therapy clinics. Indication for participation will be a need related to enhancing PF, for example tiring quickly, being quickly out of breath or being unable to keep up with peers during PA. During the treatment the participants will be engaged in a group training (2 × 60 min/week, 10 weeks) targeting cardiorespiratory fitness using high intensity interval training, muscle strength using exercises without weights and anaerobic power using plyometrics. Training intensity during high intensity interval training will be monitored with heart rate monitors, if necessary the intensity will be adjusted. Using a single-arm mixed-method design, the preliminary effectiveness will be determined using the 20 meter Shuttle Run Test, hand held dynamometry (JAMAR and MicroFET) and the Muscle Power Sprint Test, which will be assessed in week 0, 11, and 23. Feasibility will be assessed by interviewing parents and children and by organizing a focus group session with the trainers at the end of We12BFit!-PF. Based on a 5% improvement in VO_2peak_ the minimum sample size is 19 children.

**Ethics and dissemination:** The University of Groningen, University Medical Center Groningen medical ethics committee approved the study (METC 2015.216). Final results will be disseminated via scientific publications, presentations and congress proceedings. Funding organizations will receive a final study report.

**Trial Registration:** This study was registered with Netherlands Trial Registry (NTR6334, www.trialregister.nl).

## Introduction

Children with Developmental Coordination Disorder (DCD) have difficulties with coordinated motor skills, “manifested as clumsiness (e.g., dropping or bumping into objects) as well as slowness and inaccuracy of performance of motor skills (e.g., catching an object, using scissors or cutlery, handwriting, riding a bike, or participating in sports)” (1). DCD interferes significantly with daily activities and often persists into adulthood. Approximately 5–8 percent of all school children have DCD.

Likely as a consequence of their motor problems (2), children with DCD are at risk of low levels of physical fitness (PF) (3). Children with DCD have problems with all five health-related PF components: cardiorespiratory fitness (CRF) (3–6), muscle strength (3–6), muscle endurance (3), flexibility (7–9), and body composition (3–5). This deprived PF can have serious consequences on short and long term aspects of functioning and health. In the short-term, psychological and cognitive functioning may be negatively affected, whereas long-term effects may include cardiovascular disease (10). The seriousness of these consequences stresses the need for treatment of impaired PF next to treating the problems in motor coordination of children with DCD.

To our knowledge, no treatments directly targeting multiple components of health-related PF have been developed for children with DCD. However, a number of studies targeted a single component of PF. One intervention focused on endurance training for 16 weeks, three times 50 min per week, and found that children with DCD improved their CRF (11). Others have shown that it is possible to induce strength gains in children with DCD (12–14). In one study children with DCD participated in weekly Taekwondo training aimed at improving muscle strength combined with exercises to practice at home (12). After 12 weeks children with DCD had significantly improved their muscle strength, to a level comparable to typically developing (TD) children. In two case studies improvements in muscle strength were found after strength training programs of ~12 weeks (13, 14). From these studies it can be concluded that children with DCD are able to exercise and improve components of PF, despite their motor coordination problems.

In the current study a treatment will be developed targeting multiple components of PF in children with DCD. Intervention development requires a systematic method to ensure adequate design and evaluation of the treatment. Treatment theory can be used to carefully define three subsequent domains of treatment development: treatment targets and participants, mechanism of action and treatment ingredients (15). *Treatment targets* are the main aspects of functioning that are expected to be improved by the treatment and are changed *directly* by the mechanism of action of the treatment. This *mechanism of action* offers a theoretical account of how essential ingredients induce changes in the treatment target. *Treatment ingredients* are “observable (and, therefore, in principle, measurable) actions (…) that are selected or delivered by the clinician” (p. S32.e2). This includes *essential ingredients*, hypothesized to be *necessary* to induce an effect on the treatment target, and *other active ingredients* that moderate the treatment effects. Treatment ingredients can be specified by reporting dosing parameters, progression and type of activity. Defining a treatment theory compels one to explicate the underlying theory of the treatment which leads to critical and possibly more comprehensive choices in the treatment development and evaluation. In turn this allows for more detailed insight in mechanisms and effective components of treatments.

The aim of this study is to describe 1. the design and rationale of a training called We12BFit!-PF targeting cardiorespiratory fitness, muscle strength and anaerobic power in 7-12-year-old children with DCD and 2. the methods to examine its preliminary effectiveness and feasibility. We hypothesize that CRF, muscle strength and anaerobic power will improve and that the treatment will be feasible. We12BFit!-PF is the first part of a multidisciplinary treatment called We12BFit!. The second part, We12BFit!-Lifestyle physical activity, aims to improve the children's lifestyle physical activity (PA) using parent coaching, provision of information and self-monitoring of activity [described elsewhere (16)].

## Methods

### Design and Rationale of We12BFit!-PF

#### Method of Treatment Development

The development of We12BFit!-PF is based on a literature search and a focus group with professionals, which will be discussed using the three consecutive steps of defining a treatment theory: targets, mechanism of action, and ingredients.

A scoping literature search was performed in PubMed, WebOfScience and PsychInfo, including background information on DCD, theories on exercise physiology, PF treatments and reference values for PF parameters (to ascertain inclusion criteria and targets). Due to the lack of research on these topics in children with DCD, the scope was broadened to populations matching the target group as closely as possible with regard to age or impairment. This included children suspected of DCD, children with cerebral palsy, chronic diseases, typically developing children and adolescents. As the development of a treatment is a comprehensive process, we provided a narrative synthesis.

A two hour focus group was organized to establish a process of co-creation between professionals with different professional background and expertise in the field of DCD and/or training of PF. In order to stimulate the discussion and to be comprehensive we aimed to compose a heterogeneous focus group. Participants were selected to have overlapping expertise on the full range of subtopics for the development of the treatment and different levels of experience. Moreover, they were selected to include all disciplines that might be involved in the treatment. Fifteen participants were purposely selected and invited by email and by telephone if necessary, to take part in the focus group. Eight invited professionals participated, seven professionals declined the invitation because of work-related obligations. The group consisted of two (pediatric) physical therapists, a pediatric rehabilitation physician, a motor remedial teacher, a human movement scientist with expertise in exercise physiology, two researchers in (pediatric) rehabilitation and a physical education teacher working in special education. The focus group was conducted at University Medical Center Groningen, Center for Rehabilitation and was led by a researcher (Ph.D.) with ample experience in conducting qualitative research and a background in physical therapy. The focus group leader was not involved in the design of the research and had no specific experience in treating children with DCD. Five participants were (distantly) acquainted to the focus group leader. One other researcher who was involved in the development of the treatment took field notes during the focus group. The focus group guide (Appendix A) was developed by the authors. The focus group discussion was audio-taped and transcribed verbatim afterwards. Subsequently a thematic content analysis was performed. Two researchers independently performed initial coding, placed the codes in the framework of dysfunction and treatment theory and searched for subthemes among codes using Atlas-Ti 5.2 software. Subsequently, the researchers discussed their coding until they reached consensus on the coding tree (Appendix B).

For the topics targets, participants and essential ingredients information from both literature search and focus group were combined. Mechanism of action and dosing parameters were not covered in the focus group as this is mainly a theoretical topic for which literature suffices. Two parents of a child with DCD were interviewed on their preferences for the treatment (frequency, location, activities) and two children with DCD pilot tested the training activities for at least 8 weeks.

#### Intervention: Treatment Definition

##### Step 1: Targets

Information on the five health-related PF components was gathered. According to the literature CRF, measured as VO_2max_ was 7–22% lower in children with DCD compared to TD children (3, 6). Muscle strength was about 15% lower in children with DCD compared to TD children (6). However, flexibility profiles in children with DCD were heterogeneous: some children showed high levels of flexibility whereas others showed low levels of flexibility (3). Although body composition was found to be worse in the majority of articles on children with motor problems (3), this was not found in a Dutch sample of children with DCD (6). Anaerobic power is not a health-related PF component, but is an important factor to consider, since children's daily activity patterns predominantly consist of short intermittent activities with both high intensity and low to moderate intensity activities (17, 18). Children with DCD or motor learning difficulties scored 10–30% lower than TD peers on this skill-related component of PF (3).

The focus group identified CRF, muscle strength and anaerobic power as the main components of PF that should be targeted in children with DCD. They considered these three components of PF to be impaired and argued that anaerobic power is important in the ability to keep up with the intermittent play activities that children often engage in. Their concerns seemed to be related to direct functional problems rather than to health-risk associated with low CRF and muscle strength. Moreover, the focus group considered participation, quality of life and social-emotional wellbeing, as the ultimate goal of the treatment. Furthermore, they considered a request for help related to PF as a prerequisite for participation in the treatment, rendering a quantification of low PF unnecessary.

Consequently, based on the literature and focus group, the targets selected for We12BFit!-PF are CRF, muscle strength and anaerobic power. Flexibility was found to be too heterogeneous and evidence on body composition was inconsistent. Broader goals related to participation and quality of life cannot be targeted directly and should therefore be considered as distal treatment aims that may improve indirectly by training PF.

##### Step 2: Mechanism of action

The literature indicates that changes in CRF, muscle strength and anaerobic power rely on the principles of overload and specificity. The principle of overload holds that “for a training effect to occur, a system or tissue must be challenged with an intensity, duration, or frequency of exercise to which it is unaccustomed” [(19), p. 262–263]. The adaptations that occur over time abide by the principle of specificity: “the training effect is specific to the fiber types recruited, the principal energy system involved (aerobic vs. anaerobic), the velocity of contraction and the type of muscle contraction (eccentric, concentric, or isometric)” [(19), p. 262–263].

##### Step 3.a: Essential ingredients and dosing parameters

Aiming to closely follow the principle of specificity of training, the high intensity interval training (HIIT) running protocol of Baquet et al. (20) will be used to target CRF. HIIT matches the intermittent character of children's activities (17) and allows for more variation than continuous training. In order to elicit overload, the intensity of the HIIT will be based on each individuals' maximal aerobic speed (MAS) attained on the 20 meter Shuttle Run Test (20 mSRT). As the participants are likely unaccustomed to training at (near) maximal intensity, the 8 week protocol of Baquet et al. (20) will be extended to 10 weeks (week 1 and 2 of the protocol will be repeated). Training intensity should be at least 80% of maximal heart rate (21, 22). If this is not met during two consecutive sessions, running distances will be adjusted in the next training session. The protocol is expected to be feasible for groups since time intervals are the same for all participants. Distances will depend on the participants' starting levels.

Strength exercises will be performed with body weight only (no external weights) since the participants likely have low muscle strength and problems with motor coordination (23). The plank exercise will be used as it involves large muscle groups in the whole body. Exercises of increasing difficulty will be used as the level of muscle strength may vary among children with DCD (3–6). Initially the participants will perform static exercises which will be systematically extended in duration. When a variation can be sustained for a sufficient duration, the participant will continue with a more difficult variation. Eventually this includes a dynamic strength exercise: the push up. The participants will perform three sets of fifteen repetitions of this exercise at the most (24).

Plyometric exercises start with a rapid stretch of a muscle followed by rapid shortening such as jumps and will be incorporated to improve anaerobic power. As the participants likely have low anaerobic power, they will start with two sets of 10–12 jumps. This progresses systematically into higher jumps that are performed in three sets of 10–12 jumps (25). Variations of jumps on steps and over cardboards will be used.

The HIIT, strength and plyometric exercises will be offered concurrently in 60 min sessions, twice a week on non-consecutive days for 10 weeks (20, 25, 26). No negative interference effects of aerobic training to strength gains are expected in children (27). Each session will start with a warming up with low intensity exercises, after the session the participants perform a cooling down.

The focus group provided further suggestions, such as a circuit of exercises, the wheelbarrow walk and games. In addition they identified a number of concerns that need to be addressed: fatigue, pain, low muscle tone, and the heterogeneity of motor problems experienced among children with DCD. Therefore, some training recommendations from the literature were adjusted: extended duration, slower progression of training intensity and exercise complexity throughout the treatment. The training protocol will include decision rules for adjusting training intensity if a child is unable to follow the planned intensity progression. Parents indicated that they thought that training two times a week for 60 min would be feasible. Although they expressed preference for one training at the rehabilitation center/ school combined with one training session at home, they were hesitant whether they would manage to provide the training themselves. Moreover, they indicated that their child also had to perform a few exercises at home during previous physical therapy and that adherence to that was low. Therefore, training will be two times a week at the rehabilitation center/ school. Parents suggested the wheelbarrow walk, sack races, running and ball games. Pilot testing of the training showed that the training is feasible and that more games are needed to make the activities more enjoyable. Table 1 summarizes the essential ingredients and dosing parameters for targets CRF, muscle strength and anaerobic power.

**Table 1:** Targets, ingredients, and corresponding dosing parameters of We12BFit!-PF.

**OVERALL**	
Frequency	2 Sessions/week on non-consecutive days
Time	60 min
Duration	10 Weeks
Warming up	10 Min: dynamic, low intensity aerobic activities (speed ladder, bench jumps over hands, core stability exercises)
**TARGET: CARDIORESPIRATORY FITNESS**	
**Ingredient:** HIIT based on running, with active rest between sets	
**Dosing parameters**	
Intensity	- Progressive: increasing duration and MAS
	- Relative to participant's maximal exercise capacity (110–130% MAS), distance will be extended when HIIT mean heart rate (rest included) is below 80% of maximal heart rate during two consecutive sessions
Time	25 min
Type	Running
Sets*repetitions*duration, intensity	- Start: 4*(10*10 s) at 110% MAS
	- End: 1*(5*20 s) at 110% MAS + 1*(5*20 s) at 120% MAS + 2*(5*20 s) at 130% MAS
Rest intervals	- Start: Rest between runs: 10 s; rest between sets 3 min
	- End: Rest between runs: 20 s; rest between sets 3 min
**TARGET: MUSCLE STRENGTH AND ANAEROBIC POWER**	
**Ingredients:** strength and plyometric exercises	
**Dosing parameters**	
Intensity	- Progressive: exercises of increasing difficulty
	- Relative: difficulty level of the exercise depends on individual participant's starting level
Time	10 min
Type	Training with own body weight (variations of push up, jumps)
Repetitions*duration; sets*repetitions	Strength and plyometric exercises of increasing difficulty:
	- Start: strength 1*10 s; plyometric 2*(10 to 12)
	- End: strength 3*15 s; plyometric 3*(10 to 12)
Rest interval sets	During rest of the strength exercises participants perform plyometric exercises and vice versa
Cooling down	10 min: low intensity game

*MAS, maximal aerobic speed attained at 20 mSRT*.

##### Step 3.b: Other active ingredients

In order to increase compliance, the following other active ingredients will be part of the treatment. At the start of the treatment all children and parents will receive an information letter. During an intake, personal preferences, specific participant needs, eligibility criteria, and participant questions will be addressed.

We12BFit!-PF will be provided in small groups (two to six participants). Each training session will start with a short group conversation: the trainers will check how the participants felt after the previous training and how they recovered. Furthermore, the plans for the upcoming session will be discussed using a planning board. The training components will be offered in the same order each session. Exercises will focus on maximizing success and enjoyment and avoid cognitive-motor dual tasks. The exercises will be alternated by low intensity games, a variety of sports, and activities suggested by participants.

If parents are present they will be invited to watch their child during the first and last 5 min of the session and occasionally they will be invited to join the final activity during the cooling down. Pediatric physical therapists in collaboration with people with a background in psychomotor therapy or physical education will train the participants.

#### Materials and Equipment for We12BFit!-PF

During the first training all children will receive a t-shirt with the logo of We12BFit!. Materials used during the training sessions are at least: one speed ladder, one bench and an unstable surface such as Bosu Ball for warming up; ten playground cones or similar materials to demarcate the individual distances for the HIIT; two steps and two gymnastics mats for the plyometric exercises. Each study site will receive a box with a stopwatch, game materials, and game instructions that can be used during the HIIT.

### Methods for Evaluation of Effectiveness and Feasibility

#### Study Design

In this multicenter single-arm study preliminary effectiveness and feasibility will be evaluated using mixed-methods.

#### Stepwise Procedure

**Figure 1:**
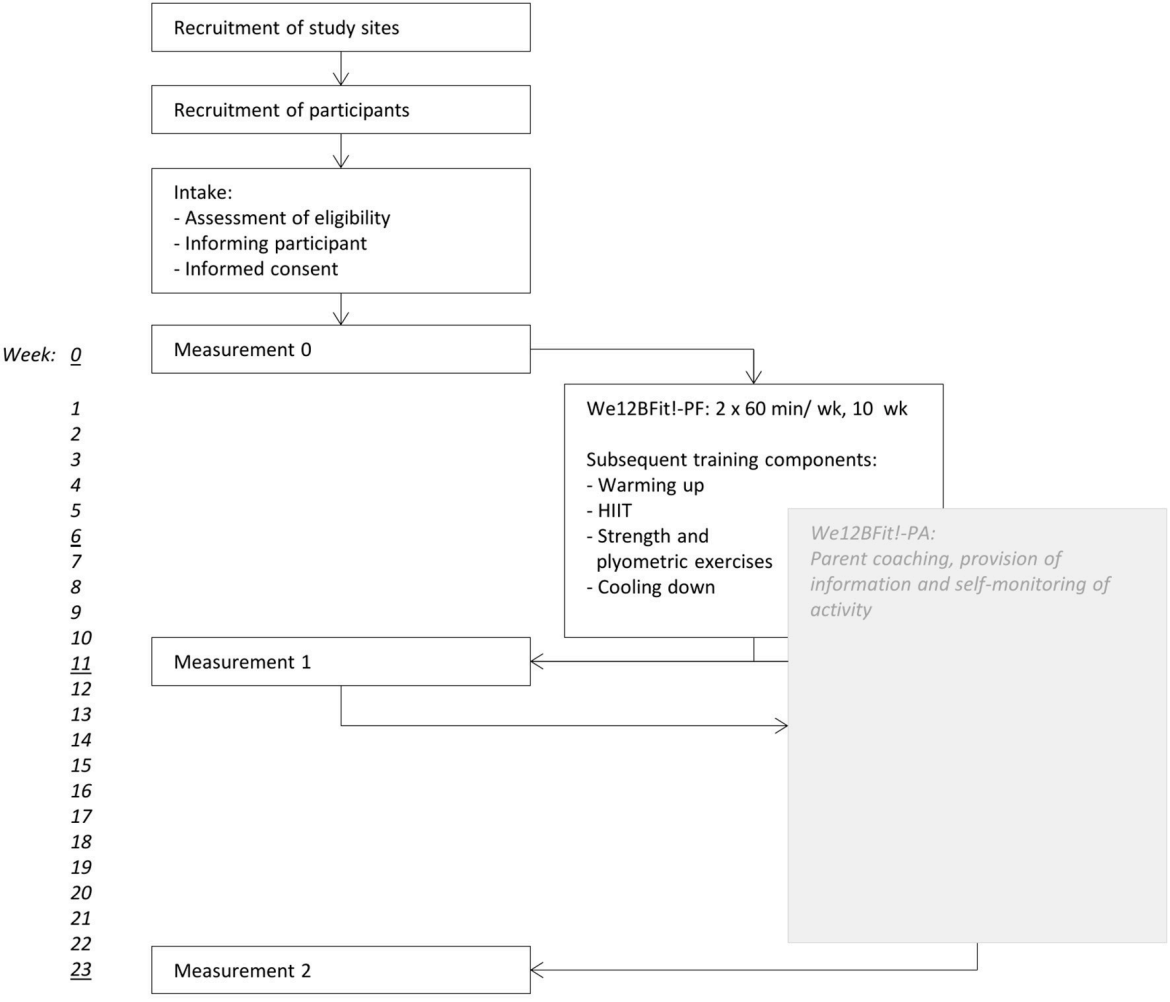
Stepwise procedure of We12BFit! and its evaluation.

Figure 1 provides an overview of the procedure that will be followed in this study.

##### Recruitment of study sites

All rehabilitation centers in the Netherlands and all pediatric physical therapy clinics in the province of Groningen will receive an invitation to participate in the study. Study site eligibility depends on willingness to participate, availability of coaches, trainers, and appropriate facilities.

##### Recruitment of participants

Physical therapists and rehabilitation physicians will be responsible for recruiting participants. Participant eligibility criteria are listed in Table 2. Participants will not partake in other physical therapy or treatments focusing on PF during their participation in We12BFit!-PF. If participants decide to withdraw their participation they will be asked for their reason to do so.

**Table 2:** In- and exclusion criteria for participation of children with DCD in the treatment.

**Inclusion criteria**
1. Previously diagnosed with DCD by a physician according to DSM-V criteria (1) or DSM-V criteria A, B and C are met and criterion D is checked in school record (pDCD).
2. Age 7–12.
3. A request for help regarding improving physical fitness. This can be operationalized as asking for help with the goal to tire less quickly, not getting out of breath as quickly anymore or stated more positively, being able to keep up with peers, being able to participate in certain activities.
4. Motivated to participate in the treatment.
5. Parents/care takers are willing to invest their time and effort.
6. It is expected that the child will benefit from the treatment (e.g., enhanced physical fitness or physical activity).
**Exclusion criteria**
1. Medical history that contra-indicates exercising or maximal exercise testing.
2. Inability to function in a group (assessed by care provider, e.g.,: the child is unable to participate in PE classes or sports activities due to social difficulties, the child disturbs activities of other children)^*^.
3. Inability to follow instructions (assessed by care provider, e.g.,: easily distracted, refuses to execute instructions, does not understand basic instructions)^*^.
4. Insufficient understanding of Dutch or English language that prevents the child to participate successfully.

* *Comorbidities such as autism spectrum disorder and attention deficit hyperactivity disorder are no reason for exclusion*.

The minimum required sample size is 19 participants. The calculation of sample size was based on the primary outcome measure VO_2peak_ (ml/ kg/ min), as attained from the 20 mSRT. First, effect size *d, d* = |x_1_ –x_2_| / (s ^*^ (1–r)^0.5^), was calculated using mean VO_2peak_ from preliminary research (x_1_) (6), mean VO_2peak_ after 5% improvement (x_2_) (21), standard deviation (s) and at least moderate Pearson correlation (*r* > 0.3). Next, sample size was calculated based on a two-tailed *t*-test with a power of 80% and alpha of 0.05, resulting in a required sample size of 19 participants.

##### Intake

When potential participants or their parents have expressed interest they will be invited for an intake with a trainer or coach of We12BFit! During the intake the trainer or coach will assess the eligibility of the child, inform the parents and their child about the intervention. If the child is eligible, the trainer or coach will provide an information letter and an informed consent. The parents and their child will be given the opportunity to ask questions, and the information letter contains the contact information of the developers of We12BFit! and of an independent physician.

##### Measurements

If parents and their child decide to participate and hand in a signed informed consent they will be scheduled for the measurements and the intervention. The first measurement will be 1 week before the first training session, the second measurement will be 1 week after the last training session and the third measurement will be 13 weeks after the last training session.

#### Anticipated Results and Materials

##### Outcome measures

The intensity of HIIT will be monitored with heart rate monitors (Polar RS300X) every training session. Preliminary effectiveness will be assessed at measurement 0, 1 and 2. The primary outcome will be the score on the 20 mSRT expressed as VO_2peak_ (ml/ kg/ min) and number of runs, indicative of CRF (28). VO_2peak_ obtained from the 20 mSRT shows moderate to good correlation with the cycle ergometer test in children scoring at the 15th percentile of the Movement Assessment Battery for Children (29). The 20 mSRT will be conducted in small groups and a therapist will join the participants for pacing and motivation. Heart rate (Polar RS300x) and the Children's OMNI Scale of Perceived Exertion will be assessed right before and after the test (30). OMNI Rating of Perceived Exertion ranges from 0 to 10 with a higher score indicating more fatigue.

Secondary measures will include handgrip strength assessed with the Jamar (Jamar-Sammons Preston, Bolingbroke, GA) hand-held dynamometer (HHD) (31), strength of elbow and knee flexion and extension assessed with the MicroFET 2 (HOGGAN Health Industries Inc, Salt Lake City, UT) HHD (32), and mean power will be assessed with the Muscle Power Sprint Test (MPST) (33). Strength measures were selected to minimally include motor coordination. The Jamar showed high reproducibility in children with myopathy (31), and is considered the gold standard for measuring hand grip strength (34). The MicroFET showed good to high reliability in children with cerebral palsy (35), and will be executed using the break method. Three measures will be taken for each muscle group of the dominant and non-dominant side. If two measures differ by more than 20%, a fourth measure will be taken. The MPST has been shown to be reliable and practical for assessing anaerobic performance in children (33). Six individual 15 m runs at maximal speed will be timed. Between runs the participants will rest for 10 s. A therapist will join the participant.

Feasibility and indirect effects of the treatment will be assessed by student observations of the training sessions. Second, parents and children will be interviewed about the treatment (Appendix C), their answers will be written down during the interview. In addition, trainers will discuss their experiences and make suggestions for improvement of the training during a two hour focus group (Appendix D). The focus group will be audio-taped and transcribed verbatim afterwards.

Potential adverse effects will be monitored both during and after the treatment. Training sessions will be observed by students using an observation scheme which includes the report of any adverse effects. In addition, the trainers will also write a report about the training sessions and during the parent interviews parents will be asked for any changes since their child's participation in We12BFit!

##### Data management

The data will be collected by researchers, who will be assisted by students. The students will enter and de-identify the data and the researchers will conduct the data analysis. Access to data will be granted only to the research team. The data will be stored securely in locked cabinets and password-protected computer files.

##### Data analysis

VO_2peak_ (ml/ kg/ min) will be calculated using the following formula: 31.025 + 3.28 ^*^ speed-3.248 ^*^ age + 0.1536 ^*^ speed ^*^ age, with speed being determined as 8 + 0.5 ^*^ final stage (28). Speed is expressed in km/ h and age in years. Strength for each muscle group will be calculated as the mean of the three closest scores. Power (Watt) will be calculated using: (body mass ^*^ 15^2^) / time^3^, with body mass expressed in kg and time in seconds (36). Mean power of the six runs will be included in the analysis. Normally distributed data will be analyzed using a dependent *t*-test. Not normally distributed data will be analyzed using a Wilcoxon test.

The focus group and interview data will be analyzed in a thematic content analysis using Atlas.ti version 8 software. Two researchers will independently code the transcript using the terminology of treatment theory and search for subthemes.

## Discussion

Although it is known that children with DCD often have lower PF than typically developing peers, no substantiated and systematically developed treatment directly targeting multiple components of PF is available for children with DCD. This study describes both the development of We12BFit!-PF, a group treatment for 7–12 year old children with DCD to improve CRF, muscle strength and anaerobic power, and the methods to evaluate the preliminary effectiveness and feasibility of this treatment.

Not every training effort automatically results in improved fitness (22). Treatment theory, as laid out in this study, may help researchers to develop viable treatments: a treatment theory compels researchers to design their treatments systematically and ensures careful selection of targets and ingredients based on the expected mechanism of action. Over time this systematic approach for developing treatments may contribute to revealing how treatments work and for whom. When applied consistently by researchers in the field this will improve study comparability, with the potential to extend our understanding of what constitutes an effective treatment.

In addition, the development of a treatment theory helps researchers to distinguish targets from aims. Aims are “aspect(s) of the (…) participant's functioning or personal factors that are predicted to change *indirectly* (…) as a result of the treatment-induced change in the treatment target” (15). Professionals in the focus group were very clear that the ultimate goal of We12BFit!-PF should be participation, quality of life and social-emotional wellbeing. These aspects are considered to be aims and not targets of the treatment. They will not be targeted directly but may improve by increased PF, or by improved social skills gained from the interactions with other children and by experiencing success during the training sessions. Moreover, training of muscle strength through strength and plyometric exercises may also improve motor skills such as running, jumping, and throwing as well and thus indirectly may improve their participation in sports (37, 38).

The aims specified by the focus group seem to reflect a durable focus on lifelong PF and participation in PA. Although we expect that PF may act as a prerequisite for engaging in PA, we expect that more factors are involved and that a more behaviorally oriented treatment is needed. Therefore we developed the complementary treatment We12BFit!-Lifestyle PA, where motivation for PA is targeted through application of behavior change strategies. It should be noted that although the designs are described separately, the effects of these treatments may not just be complementary but may also reinforce each other.

The development of this treatment was based on evidence-based findings from literature as well as experience-based and practical considerations offered by the focus group. These sources of information mainly complemented each other, but were sometimes conflicting. For instance, the focus group advocated a client-centered approach resulting in different individual treatment targets and highly tailored ingredients to enable optimal participation in PA. Although the focus group considered the group aspect to be valuable, the group aspect limited the extent to which the treatment can be adjusted to individual requests during the treatment. Moreover, the treatment theory as well as research technical considerations require predefined targets and predefined ingredients. To solve this conflict, we selected children with similar requests for help for each group and we systematically adjusted the training intensity to each child's starting level. In addition, the second part of We12BFit!, We12BFit!-Lifestyle PA, allows for a more individual approach.

There are several strengths to this study. First, an innovative aspect of this study is the use of the treatment theory allowing for thorough development of the treatment. Second, we combined information from the literature and a focus group. This resulted in both comprehensive and complementary information which informed the design of the treatment. Moreover, it allowed for the development of a treatment tailored to the needs of children with DCD, limiting the likelihood of any adverse effects such as injuries. Third, the focus group consisted of professionals with different backgrounds, ensuring that the perspectives and expertise of different professions on training of PF were included. Most experts had ample experience working with children with DCD which allowed us to direct the treatment to the specific needs of this target group. Despite these strengths a number of shortcomings should be taken into account as well. First, although we hypothesized that children with DCD are comparable to typically developing children, the information on training from the literature was on TD children and not specific to children with DCD. Testing of the treatment should reveal whether the choices made are appropriate. Second, we conducted a focus group with eight participants. In order to reach data saturation more focus groups may be required. Moreover, although parents were involved in the development of the treatment their role was limited. By means of the evaluative parent interviews after the treatment, their input in the further development of the intervention will become stronger. Finally, including a control group would improve the interpretability of the results. However, as this concerns a newly developed treatment we will not include a control group. To minimize the amount of testing for the participants no extended baseline measurements will be included in the evaluation.

To our knowledge this is the first study to describe a treatment directly targeting multiple components of PF in children with DCD. The selected treatment ingredients offer a functionally meaningful, varied and potentially enjoyable combination of activities that bear the potential of enhancing the PF of children with DCD. Evaluation of the treatment may provide insight in the trainability of PF in children with DCD. If successful, the treatment may extend the effectiveness of rehabilitation of children with DCD.

## Ethics and Dissemination

Written informed consent will obtained from parents, and children aged 12 years (Appendices E, F). The local medical ethics committee approved the study (METC 2015.216). Final results will be disseminated via scientific publications, presentations and congress proceedings.

## Author Contributions

PB, IS, FvdH and MS developed the study. PB wrote the first draft of the article. PB, IS, FvdH, CvdS, MS, and RD critically revised the paper and gave final approval for publication.

### Conflict of Interest Statement

The authors declare that the research was conducted in the absence of any commercial or financial relationships that could be construed as a potential conflict of interest.
