# Supplementary material for: We12BFit!—Improving Physical Fitness in 7–12-Year-Old Children With Developmental Coordination Disorder: Protocol of a Multicenter Single-Arm Mixed-Method Study
Source: Front Pediatr. 2018 Dec 18;6:396. doi: 10.3389/fped.2018.00396 (PMC6305409; doi:10.3389/fped.2018.00396)
Supplement: Supplementary file 1 [file Data_Sheet_1.pdf]

We12BFit! - Improving Physical Fitness in 7-12-Year-Old Children with Developmental Coordination Disorder: Protocol of a Multicenter Single-Arm Mixed-Method Study

Petra Braaksma[1]* MSc, Ilse Stuive[2] PhD, Frouwien D. van der Hoek MPST[2], Corry K. van der Sluis[1] Prof PhD, Marina M. Schoemaker[3] PhD, Rienk Dekker[1] PhD

*** Correspondence**
Petra Braaksma
p.braaksma@umcg.nl

1 Supplementary Data

APPENDIX A

Focus group topics and questions to inform the development of We12BFit!-PF

Opening question

Please briefly introduce yourself by telling who you are, where you work and what you do.

1. Treatment targets

1.1 Please write down the most important reason why it may be worthwhile to have children with DCD train their PF and the most important reason why it may not be worthwhile to have children with DCD train their PF.

1.2 What are important components of PF that children with DCD should improve?

1.3 What are feasible goals for children with DCD in a PF treatment conducted by physical therapists?

2. Recipient characteristics: in- and exclusion criteria

2.1 Please respond to the following stand: Not just children with DCD with low PF, but also children with DCD with a healthy fitness level should be invited to participate in the treatment.

2.2 How to define low PF?

3. Treatment ingredients and parameters

3.1 How do components like CRF and muscle strength relate to each other?

- Are they of equal importance?
- Would you train them simultaneously?
- Why?

3.2 How would you train the CRF of children with DCD, what activities or exercises would you recommend?

- What considerations should be taken into account when selecting these activities?

3.3 How would you train muscle strength in children with DCD, what activities or exercises would you recommend?

- What considerations should be taken into account when selecting these activities?

3.4 Is it necessary to adjust treatments for adults to children?

- If so, what adjustments would you make to a PF training when implementing it in children?
- Why?

3.5 Is it necessary to adjust CRF training for typically developing children to children with DCD?

- If so, how would you do this with regard to frequency, duration and intensity?

3.6 Is it necessary to adjust muscle strength training for typically developing children to children with DCD?

- If so, how would you do this with regard to frequency, duration and intensity?

Closure questions

Did we miss anything important?

Of all adjustments we discussed, which one is most important according to you?

APPENDIX B

Coding tree

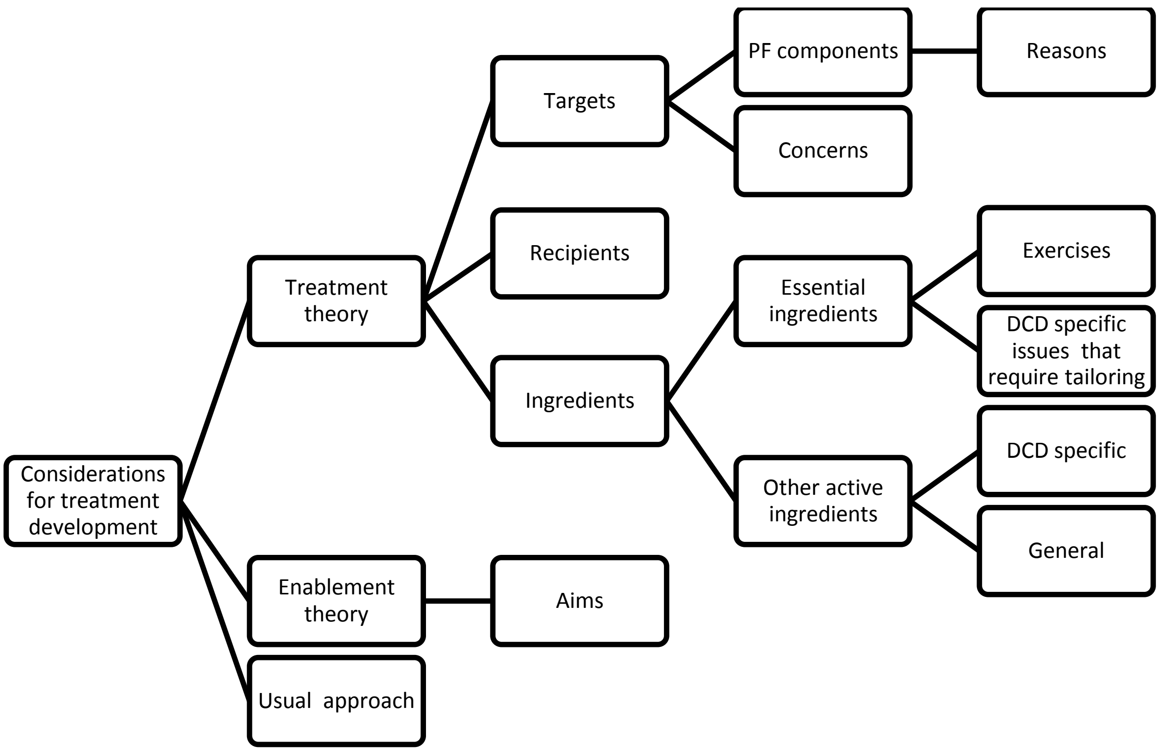

APPENDIX C

Questions for parents and children to evaluate We12BFit!-PF

**Questions for parents**	
1	How did you experience the past ten weeks during which your child participated in the training?
2	To what extent did your participation meet your expectations?
3	What did you experience as positive?
4	What did you experience as negative?
5	What would you liked to have been done differently?
6	Do you have any suggestions for improvement?
7	How do you think your child experienced the training sessions?
8	To what extent does your child understand why he/she participates in We12BFit!-PF?
9	What does your child tell you about We12BFit!-PF?
10	What does your child tell others about We12BFit!-PF?
11	What struck you about what your child told about We12BFit!-PF?
12	Have you noticed any changes since your child started We12BFit!-PF? If so, what did you notice?
13	Would you recommend other parents of a child with a similar need to participate in We12BFit!-PF? Why so or why not?
**Questions for children**	
1	Have you noticed any changes since you participated in the training sessions? If so, what did you notice?
2	What would you tell your friends about We12BFit!-PF if they were curious about it?
3	Why did you participate in We12BFit!-PF?
4	What did you like about We12BFit!-PF?
5	What didn’t you like about We12BFit!-PF?
6	How would you change the training sessions to be more fun?

APPENDIX D

Focus group topics and questions to evaluate We12BFit!-PF with the trainers

**Opening question**	
1	Please briefly introduce yourself by telling who you are and why you decided to be a trainer for We12BFit!-PF.
**Main questions**	
2	Please indicate what the participants of your training group gained from the training or what they should have improved on more according to you.<br>> How did you notice this?<br>> What did the participants and parents say about this?
3	What did you learn by providing the training?<br>> In what aspects did the training differ from your usual working method?<br>> What would you take from this training if you were to start a comparable training?<br>> What aspects of the training did you need to get used to?
4	What should be the main target of the training according to you?
5	Which bottlenecks did you come across?<br>> How to solve this?
6	What did you think of the group composition?<br>> What in- and exclusion criteria would you suggest to be adapted?
7	Which children may benefit from the training according to you?<br>> Could it be used for children with other diagnoses?<br>> How would you compose the groups if you were to include other children as well?
8	How can we accommodate the training for children with DCD with more severe behavioural problems?
9	In this set up We12BFit!-Lifestyle PA is provided by separate coaches, how do you feel about that?<br>> Would you like to contribute to We12BFit!-Lifestyle PA? If so, what would you like to do and why? If not, why not?<br>> Would you like to be a coach? If so, what do you need to able to do that? If not, why not?

**Closure questions**	
10	Did we miss anything important?
11	Of all adjustments we discussed, which one is most important according to you?

APPENDIX E

Informed consent parent/guardian

Informed consent for participation in scientific research on: ‘We12BFit!’

Statement parent/guardian:

By signing this form I declare the following:

- I read the information letter. I could ask any question I had. My questions have been answered satisfactory. I had sufficient time to decide whether I want to let my son or daughter participate in We12BFit!.

- I am aware that participation is voluntary. I am aware that I can decide to revoke my participation at any time. I am not obligated to provide an explanation for this.

- I know that the coaches and the research team of We12BFit! have access to my data that is filed for We12BFit!. I know that my data will be processed anonymously.

- I give permission to use my data for the aims listed in the information letter.

I hereby declare that I agree with the participation of my child in the abovementioned scientific research We12BFit!, on which I have been informed by an information letter.

Name parent/guardian:

Name participating child:

Signature parent/guardian: Date:

Statement researcher:
I hereby declare that I informed the abovementioned parent/guardian sufficiently about We12BFit!.

Name researcher:

Signature: Date:

APPENDIX F

Informed consent child

Informed consent for participation in scientific research on: ‘We12BFit!’

Statement child:

By signing this form I declare the following:

- I read the information letter. I could ask any question I had. My questions have been answered satisfactory. I had enough time to decide whether I want to participate in We12BFit!.

- I am aware that participation is voluntary. I am aware that I can decide to not participate anymore at any time. I am not obligated to provide an explanation for this.

- I know that the coaches and the research team of We12BFit! have access to my data that is filed for We12BFit!. I know that my data will be processed anonymously.

- I give permission to use my data for the aims listed in the information letter.

I hereby declare that I agree with the participation of my child in abovementioned scientific research We12BFit! on which I have been informed by an information letter.

Name parent/guardian:

Name participating child:

Signature participating child: Date:

Statement researcher:
I hereby declare that I informed the abovementioned participating child sufficiently about We12BFit!.

Name researcher:

Signature: Date:
